# The Gestures in 2–4-Year-Old Children With Autism Spectrum Disorder

**DOI:** 10.3389/fpsyg.2021.604542

**Published:** 2021-01-28

**Authors:** QianYing Ye, LinRu Liu, ShaoLi Lv, SanMei Cheng, HuiLin Zhu, YanTing Xu, XiaoBing Zou, HongZhu Deng

**Affiliations:** Child Development and Behavior Center, Third Affiliated Hospital of Sun Yat-sen University, Guangzhou, China

**Keywords:** autism spectrum disorder, gesture, language, adaptive behavior, social ability

## Abstract

Deficits in gestures act as early signs of impairment in social interaction (SI) and communication in children with autism spectrum disorder (ASD). However, the pieces of literature on atypical gesture patterns in ASD children are contradictory. This investigation aimed to explore the atypical gesture pattern of ASD children from the dimensions of quantity, communicative function, and integration ability; and its relationship with social ability and adaptive behavior. We used a semi-structured interactive play to evaluate gestures of 33 ASD children (24–48 months old) and 24 typically developing (TD) children (12–36 months old). And we evaluated the social ability, adaptive behavior, and productive language of ASD and TD children by using the Adaptive Behavior Assessment System version II (ABAS-II) and Chinese Communication Development Inventory (CCDI). No matter the total score of CCDI was corrected or not, the relative frequency of total gestures, behavior regulation (BR) gestures, SI gestures, and joint attention (JA) gestures of ASD children were lower than that of TD children, as well as the proportion of JA gestures. However, there was no significant group difference in the proportion of BR and SI gestures. Before adjusting for the total score of CCDI, the relative frequency of gestures without vocalization/verbalization integration and vocalization/verbalization-integrated gestures in ASD children was lower than that in TD children. However, after matching the total score of CCDI, only the relative frequency of gestures without vocalization/verbalization integration was lower. Regardless of the fact that the total score of CCDI was corrected or not, the relative frequency and the proportion of eye-gaze-integrated gestures in ASD children were lower than that in TD children. And the proportion of gestures without eye-gaze integration in ASD children was higher than that in TD children. For ASD children, the social skills score in ABAS-II was positively correlated with the relative frequency of SI gesture and eye-gaze-integrated gestures; the total score of ABAS-II was positively correlated with the relative frequency of total gestures and eye-gaze-integrated gestures. In conclusion, ASD children produce fewer gestures and have deficits in JA gestures. The deficiency of integrating eye gaze and gesture is the core deficit of ASD children’s gesture communication. Relatively, ASD children might be capable of integrating vocalization/verbalization into gestures. SI gestures and the ability to integrate gesture and eye gaze are related to social ability. The quantity of gestures and the ability to integrate gesture with eye gaze are related to adaptive behavior.

**Clinical Trial Registration:** www.ClinicalTrials.gov, identifier ChiCTR1800019679.

## Introduction

Autism spectrum disorder (ASD) is a neurodevelopmental disorder with heterogeneous manifestations mainly characterized by impairments in social interaction (SI) and communication, as well as the presence of restrictive and repetitive behaviors [Diagnostic and Statistical Manual of Mental Disorders-Fifth Edition (DSM-V); American Psychiatric Association, 2013]. The Centers for Disease Control and Prevention reported that the rate of ASD has increased to 1 in 54 (Matthew et al., 2020). Deficits and delays in gestures act as early signs of impairment in SI and communication (Lebarton and Iverson, 2016).

### Gestures in Typical Development Children

From very early in life, expressive behavior is multimodal, and early behavioral coordination is refined and strengthened for communication over time (Iverson, 2010). In the first months of life, typically developing (TD) children can communicate with others non-verbally through gestures, vocalizations, and facial expressions (Russell, 2007). It has been found that even during the prelinguistic stage, over half of the TD children can coordinate gesture and vocalization during communication (Wetherby et al., 1988). At 12 months, most TD children produce their first meaningful word (Blackwell and Baker, 2002). Before using word–word combinations, TD children start to use gesture–word combinations (Guidetti and Nicoladis, 2008; Iverson, 2010). Between 12 and 18 months, productive language and gesture are generally mutually exclusive (Capone and McGregor, 2004). At the multi-word stage, TD children use verbalization as their principal means of communication (Wetherby et al., 1988). However, from toddlers to preschoolers, combinations of gestures and verbalizations become more closely, and these cross-modal combinations can promote the development of language. At school age, gesture–speech mismatch occurs in TD children. Gesture–speech mismatch combination is a general cognitive phenomenon that reflects the transitional learning state for both science and math concepts (Capone and McGregor, 2004). In adults, communicative interactions are multimodal. They communicate with others through complex, fluid, and rapid coordination between speech, altering facial expression, changing eyebrows or head position, and gestures (Iverson, 2010).

Previous studies have found that gestures and early language development are closely linked (Bates and Dick, 2002). The development of gesture predates and predicts change in children’s language development (Iverson, 2010). For instance, Camaioni et al. (1991) found that communicative pointing at 12 months was significantly and positively correlated with vocabulary size at 20 months. And the number of different meaning gestures at 18 months predicted vocabulary at 42 months (Rowe and Goldin-Meadow, 2009). Additionally, the onset of gesture + word combinations could predict the emergence of word–word combinations (Iverson and Goldin-Meadow, 2005; Iverson et al., 2008; Iverson, 2010). Moreover, a study discovered that the number of gesture + speech combinations at 18 months predicted sentence complexity at 42 months (Rowe and Goldin-Meadow, 2009).

According to Bruner’s three earliest functions of communication, gestures can be divided into three categories: SI gestures, behavior regulation (BR) gestures, and joint attention (JA) gestures (Bruner, 1981). BR gestures are used to coordinate other people’s actions in order to make them do something or stop doing something; SI gestures are utilized to attract or keep others’ attention to oneself, with the purpose of initiating or maintaining the interaction with each other; JA gestures are used to attract others’ attention toward an event, an object, a person, or a topic, and just for showing or sharing (Bruner, 1981; Watson et al., 2013). A previous study reported that in TD children, BR gestures (at the mean age of 6.09 months) and SI gestures (at the mean age of 8.42 months) emerged before JA gestures (at the mean age of 9.33 months) (Crais et al., 2004). In general, gestures of these three communicative functions are seen in most TD children by 12 months of age (Watson et al., 2013). Wetherby et al. (1988) found that TD children displayed BR gestures and JA gestures most frequently in the second year of life.

### Gestures in Children With Autism Spectrum Disorder

Previous studies explored the quantity of gestures in children with ASD of different ages and found that the main manifestation of ASD children is the reduction of gestures (Shumway and Wetherby, 2009; So et al., 2015; Lebarton and Iverson, 2016; Özçalışkan et al., 2016). Additionally, some studies discussed the communicative function and the integration ability of gestures in ASD children.

However, the conclusions of studies about the communitive function of gestures in ASD children were not entirely consistent. In early childhood, some studies have found that ASD children used fewer gestures (i.e., pointing, showing) to direct JA compared to TD children and children with language delay (LD) (Franchini et al., 2018). Additionally, Clifford et al. (2007) detected that ASD children used proto-declarative showing less than TD children and children with developmental disorders or LD at 12–24 months. Still, there was no difference in proto-declarative pointing, social gestures, and request gestures. However, another study found that infants with autism used fewer BR gestures, SI gestures, and JA gestures than TD children and children with other developmental disabilities at 15–18 months (Watson et al., 2013). In the pre-school stage, ASD children displayed fewer JA gestures than language-matched intellectual disability (ID) children or mental-age-matched ID children and showed fewer requesting gestures than mental-age-matched ID children; however, there was no difference in SI gestures (Mundy et al., 1990). These suggest that ASD children are less likely to use JA gestures, while the use of BR gestures and SI gestures is not determined.

Previous research on the integration of gesture and other communitive means in ASD children discovered that ASD children displayed deficits in integrating gestures and vocalization/verbalization compared to TD children or LD children (Parladé and Iverson, 2015; Choi et al., 2019). However, Shumway and Wetherby (2009) found no significant difference in the proportion of total acts coordinated with a vocalization, eye gaze, and gesture at the same time between ASD children, TD children, and children with developmental delays. And Heymann et al. (2018) found that ASD children were less likely to integrate JA behaviors (including gestures and eye gaze) and vocalization than TD children. Furthermore, a study analyzed the complex level of integrating different communicative forms (gesture, vocalization, and eye gaze) and found that the level of complexity in ASD children is significantly lower than TD children (Maljaars et al., 2011). To conclude, studies on the integration of vocalization/verbalization, eye gaze, and gesture in ASD children were contradictory. Notably, when exploring the integration of gestures and eye gaze, the above studies included other communicative means, such as smile, voice, and language. Therefore, gestures which only integrated with eye gaze may be missed. The current study coded and analyzed the integration of gesture and vocalization/verbalization, and the integration of gestures and eye gaze separately. It may help us understand the cross-modal coordination ability of gestures more accurately and comprehensively for ASD children.

Recent studies showed that gesture use might also play an essential role in the development of language for ASD children. Özçalışkan et al. (2016) found that the deictic gestures of 30-month-old children with ASD could predict the vocabulary 1 year later. Besides, the emergence of distal pointing was closely linked with the emergence of first words, and the onset of gesture + word combinations predates and predicts the onset of two-word combinations (Talbott et al., 2018). In adolescence, gesture use was positively associated with vocabulary for youths with ASD (Medeiros and Winsler, 2014).

### Current Study

Previous studies have shown that the use of gestures was influenced by the variation of culture (Kita, 2009; Kwon et al., 2017). However, most of the current research works on ASD children’s gestures are based on Western-cultural conventions. This study was designed to explore the atypical gesture pattern of ASD children from the dimensions of quantity, communicative function, and integration ability based on the Chinese-cultural convention; and its relationship with social ability and adaptive behavior.

## Materials and Methods

### Participants

#### TD Group

Typically developing children were recruited through the internet or the Outpatient Department of Child Health Care at the Third Affiliated Hospital of Sun Yat-sen University in the corresponding period and met the following inclusion criteria: (1) Age 12–36 months. (2) The native language is Mandarin. (3) Parents of 12–18-month-old participants were asked to complete the Infant–Toddler Checklist (ITC; Wetherby and Prizant, 2002), and the results of the ITC must be “typical skills.” Parents of 18–36-month-old participants were asked to complete the Autism Behavior Checklist (ABC; Krug et al., 1978), and the total score of the ABC must be lower than 31. (4) All participants were confirmed as TD by two experienced developmental-behavioral pediatric specialists. The exclusion criteria are as follows: Developmental disorders, for example, ASD, ID, language disorder, etc.

#### ASD Group

All participants were recruited through the Child Developmental and Behavior Center of the Third Affiliated Hospital of Sun Yat-sen University from November 2018 to January 2020 and met the following inclusion criteria: (1) Age 24–48 months. (2) The native language is Mandarin. (3) All participants were diagnosed with ASD by two specialists in developmental-behavioral pediatrics using the Autism Diagnostic Observation Schedule (ADOS; Lord et al., 2003) and the Autism Diagnostic Interview-Revised (ADI-R; LeCouteur et al., 2003) following the DSM-V (American Psychiatric Association, 2013) criteria. The exclusion criteria are as follows: (1) Other developmental disorders, such as ID, language disorder, etc. (2) Genetic conditions associated with autism, for example, Rett syndrome, Fragile X syndrome, and tuberous sclerosis.

All participants were Chinese people. This research was approved by the Institutional Review Board at the Third Affiliated Hospital of Sun Yat-sen University and informed consent was obtained from the parents of all participants.

There are 33 children in the ASD group and 24 children in the TD group. All ASD children have received behavior intervention about 20–28 hours per week. There was no significant difference between the ASD and TD groups regarding gender, mother’s educational attainment levels, and father’s educational attainment levels. The mean age of the ASD group was older. Compared to the TD group, the scores of social skills, conceptual skills, practical skills in ABAS-II, the total score of ABAS-II, and the total score of CCDI were significantly lower in the ASD group. The characteristics and inferential statistics of participants are shown in Table 1.

**TABLE 1:** Characteristics and inferential statistics of children by group.

		ASD (*n* = 33)	TD (*n* = 24)	*df*	*χ^2^/t/Z*	*p*
		M(SD)	M(SD)			
Gender	Male	26	17	1	0.474	0.491
	Female	7	7			
Age (months)		34.18 (6.67)	23.79 (7.33)	55	5.568	< 0.001**
Mother’s Educational Attainment Levels	Bachelor degree below	14	8	2	0.940	0.625
	Bachelor degree	15	14			
	Master degree or above	4	2			
Father’s Educational Attainment Levels	Bachelor degree below	18	8	2	2.525	0.283
	Bachelor degree	13	14			
	Master degree or above	2	2			
ADOS	Communication and Social Interaction in model 1	13.92 (4.64)				
	Communication and Social Interaction in model 2	16.88 (3.83)				
ABAS-II	Social skills score	35.15 (17.12)	72.00 (17.93)	55	–7.867	< 0.001**
	Conceptual skills score	55.85 (28.21)	99.88 (44.67)	36.096	–4.251	< 0.001**
	Practical skills score	80.42 (31.62)	130.33 (51.59)	35.386	–4.200	< 0.001**
	Total score	214.61 (81.58)	351.75 (118.01)	55	–5.192	< 0.001**
CCDI	Total score	202.82 (230.14)	472.38 (364.76)		–2.917	0.004**

ADOS, Autism Diagnostic Observation Schedule; ABAS-II, Adaptive Behavior Assessment System version II; CCDI, Chinese Communication Development Inventory; M, mean; SD, standard deviation; df, degree of freedom. **p < 0.01.

### Tasks

#### Assessment of Diagnosis

In this study, the ADOS (Lord et al., 2003) and ADI-R (LeCouteur et al., 2003) were used for diagnosing ASD. We used the Chinese version of ADOS and ADI-R, which was revised by Professor Wu YuYu of Taiwan and authorized by Western Psychological Service.

#### Assessment of Communicative Gestures

A doctor who was not familiar with participants evaluated the participants’ gestural communications during a semi-structured play interaction. The content and sequence of play interaction were adapted from the ADOS. There were three main contents: blowing bubbles, blowing balloons, and snacking. Two social situations were set up in every content to encourage the children to express their demands or to show and share, while two kinds of communication opportunities. Child-initiated interaction and reactive interaction were also set up in every social situation. Only one parent was allowed to be present during the play interaction. The whole process of play interaction was videotaped for about 10 min by an assistant. The camera ensured that the child’s face and hands were recorded at the same time and the doctor’s face and hands. (The content and sequence of play interaction are shown in Supplementary Appendix 1).

#### Adaptive Behavior Assessment System Version II (ABAS-II; Oakland and Harrison, 2008)

The infant version of the Adaptive Behavior Assessment System Version II (ABAS-II) was used to assess the adaptive behavior of children aged 0–6 years. It is divided into a parent questionnaire and a teacher questionnaire. The adaptive behavior of children is evaluated from three levels. The first level is the overall adaptive function; the second level contains three composite areas of adaptive function: conceptual skills, social skills, and practical skills; the third level includes 10 concrete skill areas: communication, pre-school function, self-management, leisure, SI, community adaptability, family life, health and safety, self-care, and motor skills. Parents of participants completed the ABAS-II, which was revised by Professor Li YuQiu of Zhuhai Campus of Beijing Normal University and authorized by the American company, PEARSON. The social skills score and the total score (original score) were used to evaluate the social ability and adaptive behavior of participants. The higher the score, the better the social ability and adaptive behavior.

#### Chinese Communication Development Inventory (CCDI; Tardif et al., 2008)

Chinese Communication Development Inventory (CCDI) is the Chinese version of the MacArthur Communicative Development Inventories (MCDI; Fenson et al., 2007), which is filled out by parents. CCDI is used to assess the early language development of children aged 8–30 months who speak Chinese (Mandarin or Cantonese). CCDI can also be used to assess older children with developmental disorders. There are two forms in CCDI: the infant form (Words and Gestures) and the toddler form (Words and Sentences). We used the toddler form of the Mandarin CCDI, which is divided into two sections: productive vocabulary and sentence complexity. The total score (raw score) of these two sections was used to evaluate the productive language of participants. The higher the score, the better the productive language. The highest total score of the toddler form of Mandarin CCDI is 903 (Tardif et al., 2008). In this study, there were 23 ASD children over 30 months old; and their mean CCDI total score is 236.13, with a minimum of 0 and a maximum of 795. There were six TD children over 30 months old; their mean CCDI total score is 778.17, with a minimum of 739 and a maximum of 841. In other words, no participants who were older than 30 months had a total score of CCDI above the 50th percentile score of 30 months old (boy: 844, girl: 850; Tardif et al., 2008), and this allowed us to use the CCDI to evaluate the productive language of all participants.

### Gestures Coding

All behaviors of children in videos were coded using NVivo 12 (Windows) Pro software according to the following definitions.

#### Gestures

First of all, according to the checklist of coding gestures (Supplementary Appendix 2), we marked all target gestures of children. Second, we determined whether those gestures were used to communicate with another person (e.g., through the use of eye contact, vocalization, postural shift, repetition, or other interactive behaviors; Shumway and Wetherby, 2009; Parladé and Iverson, 2015; Özçalışkan et al., 2016). We excluded hand movements that were not used for communication. For example, we excluded imitation gestures (Braddock et al., 2015), hand movements that involved direct manipulation of an object, and hand movements that were part of a ritualized game (it should be noted that we did not exclude the showing gesture with communicative function; So et al., 2015).

#### The Communicative Function of Gestures

According to communicative function, gestures were coded using three categories (Bruner, 1981; Watson et al., 2013): (1) BR gestures are used to regulate another person’s behavior to get another person to doing something or stop doing something. (2) SI gestures are used to attract or maintain another person’s attention to oneself to initiate or maintain interaction. (3) JA gestures are used to draw another person’s attention to an object, event, person, or topic which only for sharing.

#### Integration Ability of Gestures

Temporal co-occurrence is defined as the duration of different communicative behavioral overlaps at any time point. Vocalization: children’s voices, such as vowel sound, laugh, cry, and squeal. Verbalization: single and multi-word spoken utterances. Vocalization/verbalization that was purely imitative (i.e., words repeated immediately after being spoken by another person) or not directed to another person were excluded (Shumway and Wetherby, 2009; Parladé and Iverson, 2015). With regard to any temporal co-occurrence between gesture and vocalization/verbalization, gestures were coded using these categories: vocalization/verbalization-integrated gestures and gestures without vocalization/verbalization integration.

**FIGURE 1:**
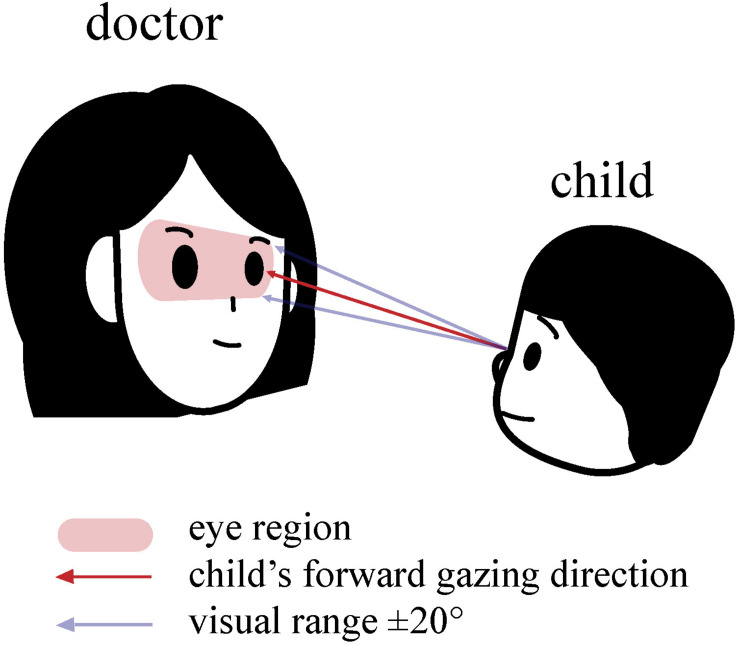
*Eye Gaze*: the visual range of the child that can intersect with the eye region of the doctor.

Eye gaze is defined as the visual attention children paid directly to another person’s eye region (Shumway and Wetherby, 2009; Parladé and Iverson, 2015). The eye region is defined as follows: In the horizontal direction, from the leftmost corner of the left eye to the rightmost corner of the right eye, and in the vertical direction, the area between the lower side of the eyebrow and the middle of the nose (He et al., 2019). The procedures of coding eye gaze: (1) Code the visual range of the child: We defined the visual range as within ± 20° of the child’s forward gazing direction. (2) Code the position in the relationship between child’s visual range and doctor’s eye region: We defined eye gaze behavior as that the visual range of the child can intersect with the eye region of the doctor (Figure 1). We defined the ±20° range based on previous research. Humans can pay visual attention to things inside the ±20° range around the facing direction, despite the direction the head faced. In contrast, they may choose to move their head when they pay visual attention to things outside of that range (Hachisu et al., 2018). Regarding whether there was any temporal co-occurrence between gesture and eye gaze, gestures were coded using these categories: eye-gaze-integrated gestures and gestures without eye-gaze integration.

### Reliability

All videos were randomly assigned to two research assistants who were blind to group allocation. Two research assistants received coding training before coding the video separately. Approximately 20% of the participant videos were randomly selected and were double coded to calculate interrater agreement. The reliability of gestures coding between the two research assistants was estimated using an intraclass correlation coefficient (ICC) using an absolute agreement definition. The ICCs for the quantity of each gestures category (single measures) were as follows: total gestures (ICC = 0.940, *p* = 0.000), BR gestures (ICC = 0.949, *p* = 0.000), SI gestures (ICC = 0.852, *p* = 0.000), JA gestures (ICC = 0.842, *p* = 0.000), gestures without vocalization/verbalization integration (ICC = 0.993, *p* = 0.001), vocalization/verbalization-integrated gestures (ICC = 0.936, *p* = 0.000), gestures without eye-gaze integration (ICC = 0.853, *p* = 0.000), and eye-gaze-integrated gestures (ICC = 0.989, *p* = 0.000).

### Research Index

We utilized the relative frequency and the proportion of gestures in each category as research indexes. The conversion method was as follows: (a) the relative frequency of gestures: dividing the quantity of gestures in each category by the duration of videos in seconds separately and then multiplying by 600 to get the rate per 10 min of gestures in each category and (b) proportion of gestures: dividing the quantity of gestures in each category by the quantity of total gestures separately to get the proportion of gestures in each category.

### Data Analysis

The analysis software used was SPSS Statistics version 23 (IBM Corp., Armonk, NY, United States), and the alpha was set at 0.05. Before conducting the data analysis, we detected the normal distribution of all data using normality tests. The total score of CCDI and some indices of gesture (the relative frequencies and the proportions of SI gestures and JA gestures, the relative frequency of eye-gaze-integrated gestures) were non-normally distributed, and the other variables were normally distributed. Therefore, Chi-square tests were conducted to explore the differences in gender and parents’ educational attainment levels between the TD and ASD groups. *T*-tests were used to analyze the differences in age and the ABAS-II between the two groups. Non-parametric statistics (Mann–Whitney tests) were utilized to explore the differences in the CCDI between the two groups. After the logarithm transformations of the gestures indexes with non-normal distribution, analysis of variance (ANOVA) and analysis of covariance (ANCOVA, corrected for the total score of CCDI) were used to explore the differences in gestures between ASD and TD groups. Pearson correlation analysis was utilized to test the correlation between gestures, social ability, and adaptive behavior. Furthermore, partial correlation analysis was used to control the total score of the CCDI to study the associations between gestures, social ability, and adaptive behavior.

## Results

Considering that there were differences in the total score of CCDI between the ASD group and TD group, we use both ANOVA and ANCOVA (corrected for the total score of CCDI) when exploring the differences in gestures between groups.

### The Quantity of Gestures

Whether we use ANOVA [*F*^1^_(__1_,_56__)_ = 43.2801, *p*^1^ < 0.001] or ANCOVA [corrected for the total score of CCDI, *F*^2^_(1,55)_ = 26.841, *p*^2^ < 0.001], we found that the relative frequency of total gestures in ASD group was lower than that in TD group (Table 2).

**TABLE 2:** Descriptive and inferential statistics for gestures of children by group.

			ASD (*n* = 25)	TD (*n* = 12)	*df*^1^	*F*^1^	*p*^1^	*df*^2^	*F*^2^	*p*^2^
			M(SD)	M(SD)						
**Quantity**	Relative frequency	Total gestures	21.90 (8.07)	38.45 (10.94)	1,56	43.280	<0.001**	1,55	26.841	<0.001**
**Communicative function**	Relative frequency	BR gestures	16.54 (5.19)	28.54 (10.30)	1,56	33.346	<0.001**	1,55	20.136	<0.001**
		SI gestures	2.66 (2.35)	4.38 (2.56)	1,56	9.453	0.003**	1,55	4.451	0.040*
		JA gestures	2.73 (3.72)	5.52 (2.98)	1,56	17.111	<0.001**	1,55	10.083	0.002**
	Proportion (%)	BR gestures	78.21 (15.76)	72.77 (10.39)	1,56	2.167	0.147	1,55	0.989	0.325
		SI gestures	11.56 (8.50)	11.81 (7.02)	1,56	1.088	0.302	1,55	0.248	0.621
		JA gestures	10.23 (11.28)	15.42 (9.65)	1,56	8.416	0.005**	1,55	4.913	0.031*
**Integration ability**	Relative frequency	Gestures without vocalization/verbalization integration	10.05 (5.69)	20.21 (7.95)	1,56	31.710	<0.001**	1,55	22.644	<0.001**
		Vocalization/verbalization-integrated gestures	11.84 (7.11)	18.15 (12.23)	1,56	6.022	0.017*	1,55	2.117	0.151
		Gestures without eye-gaze integration	12.22 (5.09)	11.90 (5.25)	1,56	0.054	0.816	1,55	0.057	0.813
		Eye-gaze-integrated gestures	9.37 (6.73)	26.55 (10.25)	1,56	41.840	<0.001**	1,55	26.581	<0.001**
	Proportion (%)	Gestures without vocalization/verbalization integration	46.28 (22.73)	55.75 (22.81)	1,56	2.401	0.127	1,55	3.052	0.086
		Vocalization/verbalization-integrated gestures	53.72 (22.72)	44.25 (22.81)	1,56	2.406	0.127	1,55	3.057	0.086
		Gestures without eye-gaze integration	58.52 (21.10)	31.82 (14.19)	1,56	28.864	<0.001**	1,55	19.726	<0.001**
		Eye-gaze-integrated gestures	41.48 (21.10)	68.18 (14.19)	1,56	28.864	<0.001**	1,55	19.726	<0.001**

BR, behavior regulation; SI, social interaction; JA, joint attention; M, mean; SD, standard deviation; df, degree of freedom. ^1^Analysis of variance. ^2^Analysis of covariance corrected for the total score of CCDI. *0.01 < p < 0.05, **p < 0.01.

### The Communicative Function of Gestures

**FIGURE 2:**
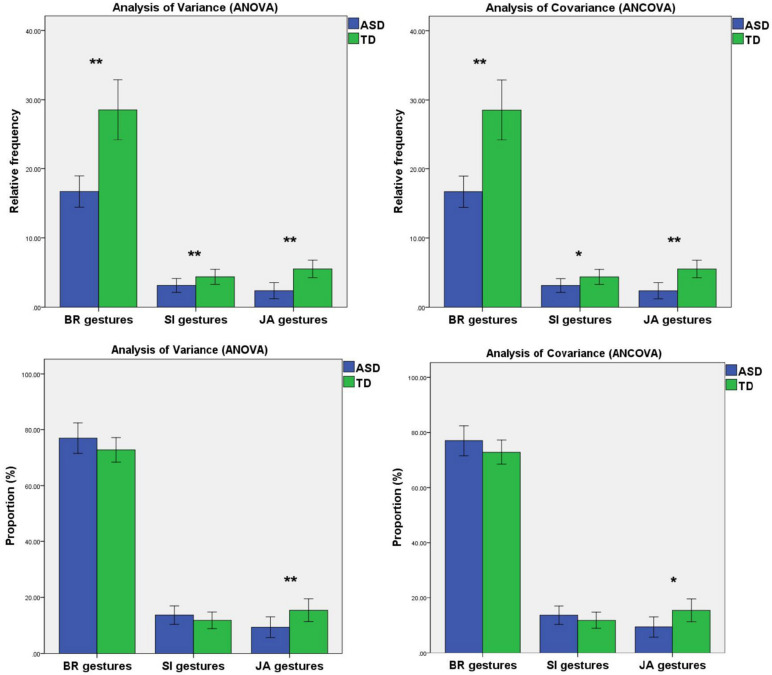
Mean relative frequency and mean proportion of BR gestures, SI gestures, and JA gestures produced by TD children and ASD children. Error bars represent standard error. *Note. BR, behavior regulation; SI, social interaction; JA, joint attention.* *0.01 < *p* < 0.05, **p < 0.01.

Whether we use ANOVA or ANCOVA, we found that the relative frequency of BR gestures [*F*^1^_(1,56)_ = 33.346, *p*^1^ < 0.001; *F*^2^_(1,55)_ = 20.136, *p*^2^ < 0.001], the relative frequency of SI gestures [*F*^1^_(1,56)_ = 9.453, *p*^1^ = 0.003; *F*^2^_(1,55)_ = 4.451, *p* = 0.040], the relative frequency [*F*^1^_(1,56)_ = 17.111, *p*^1^ < 0.001; *F*^2^_(1,55)_ = 10.083, *p*^2^ = 0.002], and the proportion [*F*^1^_(1,56)_ = 8.416, *p*^1^ = 0.005; *F*^2^_(1,55)_ = 4.913, *p*^2^ = 0.031] of JA gestures in the ASD group were significantly lower than those in the TD group, while there was no significant difference in the proportion of BR gestures and SI gestures among groups (*p* > 0.05) (Table 2 and Figure 2).

### The Integration Ability of Gestures

We found that the relative frequency of gestures without vocalization/verbalization integration [*F*^1^_(__1_,_56__)_ = 31.710, *p*^1^ < 0.001] and the relative frequency of vocalization/verbalization-integrated gestures [*F*^1^_(1,56)_ = 6.022, *p*^1^ = 0.017] in ASD group were significantly lower than that in TD group when we used ANOVA. While we utilized ANCOVA to adjust for the total score of CCDI, we found that only the relative frequency of gestures without vocalization/verbalization integration was significantly lower [*F*^2^_(1,55)_ = 22.644, *p*^2^ < 0.001]. Moreover, when we used ANOVA or ANCOVA, we found no significant difference among ASD and TD groups in the proportion of vocalization/verbalization-integrated gestures and gestures without vocalization/verbalization (*p* > 0.05).

**FIGURE 3:**
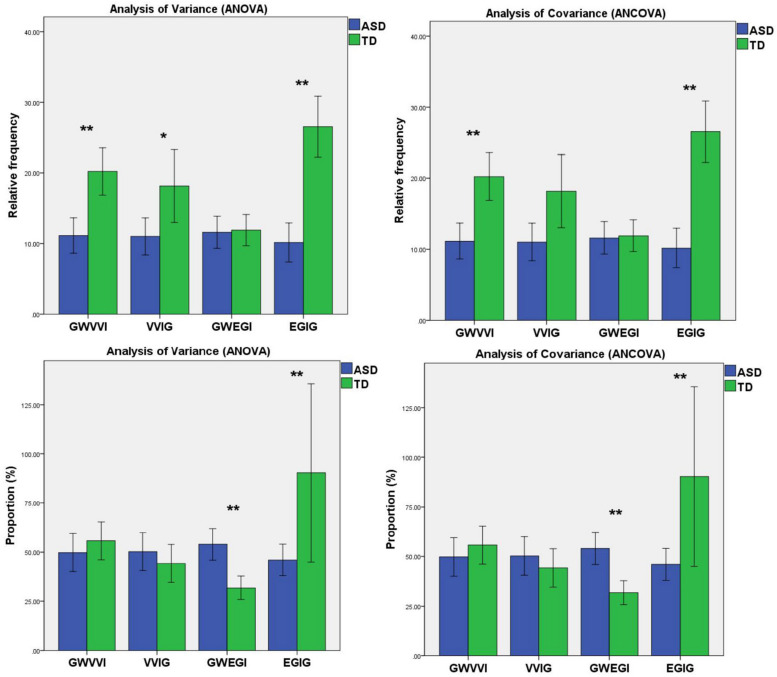
Mean relative frequency and mean proportion of gestures without vocalization/verbalization integration, vocalization/verbalization-integrated gestures, gestures without eye-gaze integration, and eye-gaze-integrated gestures produced by TD children and ASD children. Error bars represent standard error. *Note. GWVVI, gestures without vocalization/verbalization integration; VVIG, vocalization/verbalization-integrated gestures; GWEGI, gestures without eye-gaze integration; EGIG, eye-gaze-integrated gestures.* *0.01 < *p* < 0.05, **p < 0.01.

Regardless of utilizing ANOVA or ANCOVA, we found that the relative frequency of eye-gaze-integrated gesture [*F*^1^_(__1_,_56__)_ = 41.840, *p*^1^ < 0.001; *F*^2^_(1,55)_ = 26.581, *p*^2^ < 0.001] and the proportion of eye-gaze-integrated gesture [*F*^1^_(1,56)_ = 28.864, *p*^1^ < 0.001; *F*^2^_(1,55)_ = 19.726, *p*^2^ < 0.001] in the ASD group were significantly lower than that in TD group. And ASD group showed a higher proportion in gestures without eye-gaze integration than the TD group [*F*^1^_(1,56)_ = 28.864, *p*^1^ < 0.001; *F*^2^_(1,55)_ = 19.726, *p*^2^ < 0.001]. Besides, we found no significant difference among ASD and TD groups in the relative frequency of gestures without eye-gaze integration (*p* > 0.05) (Table 2 and Figure 3).

### The Relationship Between Gestures and ABAS-II

We found some statistically different gestural indexes between ASD and TD groups by utilizing ANCOVA. Those indexes might reflect the deficiency of ASD children’s gestural communications most. Therefore, for reducing the number of calculated correlations, we only analyzed the correlation between ABAS-II and those gestural indexes. Besides, considering that the “proportion” indexes (i.e., the proportion of gestures without eye-gaze integration and the proportion of eye-gaze-integrated gestures) are merely complementary to each other, we only choose the proportion of eye-gaze-integrated gestures.

In ASD group, social skills score in ABAS-II was positively correlated with the relative frequency of SI gestures (*r* = 0.368, *p* = 0.035) and eye-gaze-integrated gestures (*r* = 0.375, *p* = 0.032); the total score of ABAS-II was positively correlated with the relative frequency of total gestures (*r* = 0.401, *p* = 0.021) and eye-gaze-integrated gestures (*r* = 0.411, *p* = 0.017). In TD group, the scores of ABAS-II were not significantly correlated with gestures (*p* > 0.05 for all) (Table 3).

**TABLE 3:** Correlation between gestures and ABAS-II.

			Social skills score in ABAS-II				Total score of ABAS-II			
			ASD (*n* = 33)		TD (*n* = 24)		ASD (*n* = 33)		TD (*n* = 24)	
			*r*	*P*	*r*	*P*	*r*	*p*	*r*	*p*
Quantity	Relative frequency	Total gestures	0.328	0.062	0.322	0.125	0.401	0.021*	0.305	0.148
Communicative function	Relative frequency	BR gestures	0.266	0.135	0.201	0.347	0.339	0.054	0.211	0.323
		SI gestures	0.368	0.035*	0.262	0.216	0.315	0.074	0.282	0.182
		JA gestures	0.223	0.213	0.371	0.075	0.304	0.085	0.231	0.278
	Proportion (%)	JA gestures	0.167	0.354	0.245	0.249	0.229	0.141	0.128	0.551
Integration ability	Relative frequency	Gestures without vocalization/verbalization integration	0.100	0.578	0.037	0.864	0.203	0.200	0.128	0.550
		Eye-gaze-integrated gestures	0.375	0.032*	0.364	0.080	0.411	0.017*	0.274	0.194
	Proportion (%)	Eye-gaze-integrated gestures	0.065	0.721	0.027	0.899	0.098	0.586	0.010	0.965

BR, behavior regulation; SI, social interaction; JA, joint attention; ABAS-II, Adaptive Behavior Assessment System version II; r, related coefficient. *0.01 < p < 0.05.

When we controlled the total score of CCDI, the scores of ABAS-II were not significantly correlated with gestures in ASD group (*p* > 0.05 for all). In TD group, the social skills score in ABAS-II was positively correlated with the relative frequency of JA gestures (*r* = 0.439, *p* = 0.036) when controlling the total score of CCDI (Table 4).

**TABLE 4:** Partial correlation between gestures and ABAS-II.

			Social skills score in ABAS-II				Total score of ABAS-II			
			ASD (*n* = 33)		TD (*n* = 24)		ASD (*n* = 33)		TD (*n* = 24)	
			*r*	*p*	*r*	*P*	*r*	*p*	*r*	*p*
Quantity	Relative frequency	Total gestures	0.283	0.117	0.096	0.662	0.334	0.062	0.013	0.954
Communicative function	Relative frequency	BR gestures	0.253	0.162	–0.058	0.794	0.342	0.055	–0.116	0.597
		SI gestures	0.324	0.071	0.145	0.510	0.228	0.210	0.156	0.477
		JA gestures	0.145	0.427	0.439	0.036*	0.164	0.369	0.291	0.177
	Proportion (%)	JA gestures	0.095	0.605	0.389	0.066	0.098	0.594	0.289	0.181
Integration ability	Relative frequency	Gestures without vocalization/verbalization integration	0.113	0.536	–0.114	0.604	0.253	0.163	–0.026	0.906
		Eye-gaze-integrated gestures	0.321	0.073	0.171	0.436	0.317	0.078	–0.013	0.954
	Proportion (%)	Eye-gaze-integrated gestures	0.029	0.876	–0.120	0.587	0.034	0.855	–0.194	0.376

BR, behavior regulation; SI, social interaction; JA, joint attention; ABAS-II, Adaptive Behavior Assessment System version II. r, related coefficient. *0.01 < p < 0.05.

## Discussion

The present study aimed to explore the differences in gestures between ASD children and TD children in different productive language levels. As expected, we found an atypical gestures pattern of ASD children from the dimensions of quantity, communicative function, and integration ability.

### The Quantity of Gestures

We found that ASD children had lower scores of CCDI compared to TD children. And no matter that we corrected for the total score of CCDI or not, we found that ASD children displayed fewer gestures than TD children. ASD children have impairments in SI and social communication, and their communication deficits are not limited to spoken language but also gesture (Iverson et al., 2017). In other words, in the early development of life, ASD children produce fewer gestures than TD children regardless of their productive language. Similarly, Mastrogiuseppe et al. (2015) found that the quantity of gestures produced by ASD children (chronological age range 30–66 months) was significantly lower than in TD children.

### The Communicative Function of Gestures

Before and after controlling for the total score of CCDI, we found that ASD children used less BR, SI, and JA gestures than TD children, and the proportion of JA gestures in ASD children was significantly lower. The differences in the relative frequencies of BR, SI, and JA gestures between ASD and TD groups might be due to the overall differences in gesture productions. And the possible explanation for the lower proportion of JA gestures is that JA gestures are related to more complex triadic interactions. For example, ASD children might need to coordinate attention between themselves, the doctor, and objects/location/event at the same time when using JA gestures (e.g., pointing to the bubble to let the doctor notice the bubble’s location). In TD children, dyadic interaction with another person forms in the first 3 months of life, and dyadic interaction with object forms in the first 6 months of life. At 9–12 months, TD children begin to coordinate the two types of dyadic interactions to form triadic interactions (Bard, 2016). These complexities of triadic interactions might explain the deficits of ASD children in the use of JA gestures. Other studies have reported reduced triadic gestures in ASD children. For example, Watson et al. (2013) coded retrospective home videotapes and found that ASD children use fewer JA gestures at 9–12 and 15–19 months. In a prospective study of infant siblings at high-risk and low-risk ASD infants, Franchini et al. (2018) reported that initiations of JA were impaired from 12 months in ASD children, especially in the use of gestures (i.e., showing and pointing). Significantly, there was no difference in the proportion of SI gestures between ASD and TD children. One interpretation of this finding is that the limited semi-structured play situations might not have effectively triggered SI gestures from the children. Therefore, we may have underestimated the use of SI gestures in TD children.

### The Integration Ability of Gestures

Before adjusting for the total score of CCDI, we found that ASD children were less likely to integrate gesture and vocalization/verbalization than TD children. However, after correcting for the total score of CCDI, we found that the ability of ASD children to integrate gesture and vocalization/verbalization was no different from TD children. Recently, Murillo et al. (2020) found that there is no difference in the proportion of gesture + vocalization combinations between ASD children and language-matched TD children. Previous studies have shown that gestures will be combined with speech temporally and semantically when children enter the two-word stage of language development (Sowden et al., 2008). This suggests that the development of spoken language and gestures is concurrent. From the results of this study, we believe the reduction in the integration of gesture and vocalization/verbalization may merely be a potential sign of LD. ASD children might be capable of integrating vocalization/verbalization with gestures.

Conversely, no matter that we corrected for the total score of CCDI or not, ASD children were worse at integrating gesture and eye gaze than TD children. Likewise, Murillo et al. (2020) suggested that ASD children did not integrate gaze with gestures as TD children did, regardless of their productive vocabulary. This indicates that eye gaze is closely related to the functional use (i.e., integration ability) of gestures. Previous studies have found that individuals with ASD have already experienced difficulties in social orientation in their infancy. Compared to TD individuals, individuals with ASD demonstrate decreased attention to socially relevant stimuli. In particular, they have a deficiency in processing the facial information of other people, as well as in establishing and maintaining eye contact (Guillon et al., 2014). We believe that the deficiency in the ability to integrate gestures with eye gaze seen in ASD children might be the core feature of their social impairment on the level of gestures.

### The Relationship Between Gestures and Social Ability

For ASD children, the better social ability, the more SI gestures, and the better ability to integrate gesture and eye gaze. SI gestures are used to attract or maintain another person’s attention to oneself to initiate or maintain interaction (Watson et al., 2013). Thus, we decided that in terms of gestures, communication by SI gestures manifests better social ability in ASD children. Besides, previous studies have found that ASD children had impairment in facial perception; they reduced fixation on faces and eye region (Klin et al., 2002; Rice et al., 2012). The ability of facial perception of ASD children was related to their social ability (Klin et al., 2002; Mcpartland et al., 2011; Parish-Morris et al., 2013). Combined with the above results, we believe that the ability to integrate gesture and eye gaze in ASD children might be able to reflect their social ability.

### The Relationship Between Gestures and Adaptive Behavior

Previous studies have shown that ASD children had deficits in adaptive behavior (Mouga et al., 2015; Bradshaw et al., 2018). The improvement of adaptive behavior is one of the crucial outcomes of ASD intervention (Zachor and Ben-Itzchak, 2017). However, only a few studies preliminary discussed the relationship between adaptive behavior and gestures. For example, Kjellmer et al. (2012) found that for ASD children, non-verbal communications seem to be related to adaptive behavior, and Stamper et al. (2010) found that ASD children’s deficits in gestural communication are related to adaptive behavior. Importantly, this study explored the relationship between gestures and adaptive behavior from the aspects of gestures’ quantity, gestures’ communicative function, and gestures’ integration ability. The results showed that in ASD children, the number of gestures and the ability to integrate gesture and eye gaze positively correlate with adaptive behavior. It is probably because when ASD children produce more gestures, they communicate with others more. Furthermore, when communicating by gestures, eye-gaze integration may make communicative behavior more natural and smooth so that ASD children can better adapt to social life.

However, when controlling the productive language, the correlations between gesture, adaptive behavior, and social ability disappeared in ASD children. It may indicate that the relationships between gesture, adaptive behavior, and social ability are influenced by productive language. Future studies should explore the role productive language plays on relationships between gesture, social ability, and adaptive behavior.

In the TD group, we found no significant relationship between gestures, social ability, and adaptive behavior. However, when we controlled productive language, the social ability was positively correlated with JA gestures. That is probably because the development of gestures in TD children is more closely related to language development (Goldin-Meadow and Alibali, 2013).

### Limitation

By reviewing research, we can find that TD children’s gestures and language develop rapidly in the second and third years after birth. Consequently, we enrolled 12–36 months TD children. And gestures and early language development are closely linked. It is necessary to consider the impact of language when exploring the difference in gesture patterns between ASD children and TD children. In some previous research on gestures of ASD children, the chronological age of TD children ranged from 12 to 36 months. After matching, they enrolled ASD children who are 1–2 years older than TD children (Mastrogiuseppe et al., 2015; Özçalışkan et al., 2016, 2017). According to these research works, we enrolled 24–48 months ASD children to make the productive language between ASD children and TD children more comparable. In future work, we should use relevant assessments to match productive language development between the ASD and control groups. The semi-structured play situation may elicit communication strategies that are not operated by ASD children in naturalistic situations. In the future, we can investigate the pattern of ASD children’s gestures in natural situations by coding family videos. Moreover, there are only TD children in the control group of this study. Children with other developmental disorders need to be included in future studies to ensure that the results are more specific. Last, in the associations between gestures and ABAS-II, the significance level around 0.01 < *P* < 0.05, which might be a consequence of the Type 1 error chance. In future studies, we should increase the number of participants and set up the significance level of *p* < 0.01.

## Conclusion

We discovered the atypical gesture patterns of ASD children: (1) ASD children produce fewer gestures and have deficits in triadic interaction gestures (i.e., JA gestures). (2) The deficiency of integrating eye gaze and gesture is the core deficit of ASD children’s gesture communication. Relatively, children with ASD might be capable of integrating vocalization/verbalization into gestures. Furthermore, we found that SI gestures and the ability to integrate gestures and eye gaze are related to the social ability. The quantity of gestures and the ability to integrate gestures with eye gaze are related to adaptive behavior.

## Data Availability Statement

The raw data supporting the conclusions of this article will be made available by the authors, without undue reservation.

## Ethics Statement

The studies involving human participants were reviewed and approved by the Medical Ethics Committee of the Third Affiliated Hospital of Sun Yat-sen University. Written informed consent to participate in this study was provided by the participants’ legal guardian/next of kin.

## Author Contributions

QY, XZ, and HD contributed to conception and design of the study. QY and SC organized the database. QY, LL, and HZ performed the statistical analysis. QY wrote the first draft of the manuscript. QY, SL, YX, and HD wrote the sections of the manuscript. All authors contributed to manuscript revision, and read and approved the submitted version.

## Conflict of Interest

The authors declare that the research was conducted in the absence of any commercial or financial relationships that could be construed as a potential conflict of interest.
