# Supplementary material for: The Gestures in 2–4-Year-Old Children With Autism Spectrum Disorder
Source: Front Psychol. 2021 Jan 28;12:604542. doi: 10.3389/fpsyg.2021.604542 (PMC7875888; doi:10.3389/fpsyg.2021.604542)
Supplement: Supplementary file 1 [file Table_1.docx]

**Supporting Information**

**Appendix 1**

*The Content and Sequence of Play Interaction*

| Preparation before assessment | After entering the room, the doctor lets the child play freely to adapt to the room, for a pleasant and relaxed atmosphere; and invites the child to sit down after the child has relieved his/her tension. The doctor sits opposite the child, and one of the parents sits on the child's right side. The doctor tells the parents. "I want to play a series of games with your child. I want to see what the child will do without your guidance and intervention. If I need your help, I will tell you. Thank you." Then, start the assessment and video recording. |
| --- | --- |
| Blowing bubbles | 1. The doctor takes out the bubble jar and puts it about 50cm in front of the child. Then the doctor blows bubbles for 5 seconds to see whether the child pays attention to the bubbles.  2. Set up a situation to encourage the child to express his/her demands initiatively: The doctor closes the cover of the jar and puts the bubble jar on the table to wait for the child to ask for the bubbles to be blown again.  3. Set up a situation to encourage the child to express his/her demand passively: The doctor blows bubbles and raises the bubble jar so that the child can see it but cannot reach it, then asks the child, "Do you want it?" and waits for the child to respond.  4. Set up a situation to encourage the child to show and share initiatively: The doctor blows bubbles and alternates gaze between bubbles and the child at the same time to let the child pay attention to bubbles, and waits for the child to show and share on his/her initiative.  5. Set up a situation to encourage the child to show and share passively: The doctor blows bubbles and alternates gaze between the bubbles and the child. Then the doctor asks, "Where are the bubbles?" to wait for the child to respond. |
| Blowing balloons | 1. The doctor blows up the balloon slowly and exaggeratedly and squeezes the mouth of the balloon to prevent air leakage. Then the doctor puts the balloon in front of the child to let the child touch it, and says, "1, 2, 3, fly." Simultaneously, the doctor releases the balloon to let it fly for a distance in the room to see whether the child pays attention to the balloon.  2. Set up a situation to encourage the child to express his/her demand initiatively: The doctor blows up the balloon and squeezes the mouth of the balloon. Then the doctor says, "1, 2, 3, fly," and releases the balloon to let it fly for a distance in the room. The balloon lands within the child's sight. The doctor waits for the child to pick up the balloon to ask for the balloon to be blown again.  3. Set up a situation to encourage the child to express his/her demand passively: The doctor blows up the balloon and squeezes the mouth of the balloon. Then the doctor raises the balloon so that the child can see the balloon but cannot touch it. The doctor asks, "Do you want the balloon?" and waits for the child to respond.  4. Set up a situation to encourage the child to show and share initiatively: The doctor blows up the balloon and squeezes the mouth of the balloon. Then the doctor says, "1, 2, 3, fly," and releases the balloon to let it fly for a distance in the room. The balloon lands within the child's sight. Next, the doctor alternates his/her gaze between the balloon and the child to let the child pay attention to the balloon and waits for the child to show and share on his/her initiative.  5. Set up a situation to encourage the child to show and share passively: The doctor blows up the balloon and squeezes the mouth of the balloon. Then the doctor says, "1, 2, 3, fly," and releases the balloon to let it fly for a distance in the room. The balloon lands within the child's sight. Next, the doctor asks, "Where is the balloon?" and waits for the child to respond. |
| Snacking | 1. The doctor invites the child to sit down and says, "It is snack time now."  2. Set up a situation to encourage the child to express his/her demand initiatively: The doctor puts two kinds of snacks that children like to eat in two transparent boxes respectively, and says, "I have biscuits and chips". The doctor gives a piece of each snack to the child, then closes the lids of boxes and puts these two boxes on the table to wait for the child to ask for more snacks.  3. Set up a situation to encourage the child to express his/her demand passively: The doctor raises the boxes so that the child can see them but cannot touch them and asks, "Which one do you want?" or "Would you like some biscuits?" and waits for the child to respond.  4. Set up a situation to encourage the child to show and share initiatively: The doctor puts some snacks on the lid and gives them to the child, waiting for the child to share the snacks on his/her initiative.  5. Set up a situation to encourage the child to show and share passively: The doctor puts some snacks on the lid and gives them to the child. Then the doctor asks, "I want to eat biscuits, can I have some?" and waits for the child to respond. |
| Ending | The doctor put away the toys and snacks and says, "Game is over, goodbye." |
| Matters needing attention | Two social situations were set up in every content to encourage the child to express his/her demands or to show and share, while two kinds of communication opportunities, a child-initiated interaction and a reactive interaction were also set up in each social situation. ALL communication opportunities in the social situations were set with a 3-second waiting time. If the children responded appropriately, the assessment moved on to the next communication opportunity or social situation. If the child did not respond, the doctor repeated the prompts. The maximum number of prompts was set at 3. |

**Appendix 2**

*The Checklist of Coding Gestures*

| Coding gesture | Definition |
| --- | --- |
| Reach | With minimal body movement, put out one's hand or arm to reach for something that can't be reached. If you try to move your body to reach for it, the gesture will not be recorded. |
| Nod | Moving head up and down to express "yes", "want to", "ok", "agree", "greet", and so on. |
| Shake head | Shaking head left and right to express "no", "I don't want", "I can't", "disagree", "refuse", and so on. |
| Point | Extend the index finger while the other four fingers are retracted. The index finger is pointing to an object |
| Give | Put the object in your hand toward another person or put the object in your hand on another person's palm. |
| Show | Raise something toward another person and keep it stationary for 1-2 seconds, or wave something toward someone to attracting their attention but not for asking for help. |
| Open palm | Put out the hand(s) with palm(s) turned upward and ask for something out of reach. |
| Pull | Pull another person toward oneself by touching another person’s body (e.g., hand, arm, trunk) or clothes in order to attract the person's attention. |
| Pat | Patting another person’s body such as hand, arm, or torso with your hand to attract attention, but not as an aggressive behavior. |
| Push | Push others away with a hand to tell others the meaning of “no”, “get away from me”, etc. |
| Raise hand for a hug | Raise hands towards others in order to be picked up. |
| Applaud | Palms clap at each other to express “I am happy", "That’s great", etc. |
| Wave hand | Wave a hand or arm to express "goodbye". |
| Swing hand | Swing hand up and down in order to draw others' attention and get them close to you. |
| Blow a kiss | Put your hand on your lip, and then slowly move your hands away from your lips, gradually reaching out to other people. The meaning is "goodbye". |
| Make ”Shh” | Hold out the index finger, put it on the lips, and make an "Shh" sound to keep others quiet. |
| Shake hand | Shaking hand or arm left and right to express “no”, “don’t”, ”reject”, etc. |
| Spread hands | Put out two hands with an upturned palm to express “nothing”, ”something is gone”. |
| Make ”YEAH” | Hold out the index and middle fingers, with the other three fingers are retracted. The meaning can be “happy”, ”That’s great”, etc. |
| Thumb up | Thumb up and the other four fingers retracted to indicate "great". |
| Iconic gestures | Iconic gestures depicted the attributes or actions of an object through hand or body movements (Lebarton & Iverson, 2016), for example, flapping arms to convey ‘bird’. |
| Other gestures | Other gestures that meet the definition of gestures and that are utilized to communicate with other people. |
